# International collaboration in the Nursing agenda in the coming decades

**DOI:** 10.1590/1518-8345.0000.2739

**Published:** 2016-11-21

**Authors:** Maria Cecilia Bueno Jayme Gallani

**Affiliations:** Associate Editor of the Revista Latino-Americana de Enfermagem and Full Professor of the Faculté des Sciences Infirmières, Université Laval, Québec, Canada. E-mail: maria-cecilia.gallani@fsi.ulaval.ca

**Figure:**
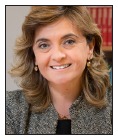

The society requires from healthcare professionals the responsibility for developing, maintaining and optimizing both the quality of care provided to citizens and the health system integrity as a whole. The frequent changing in health context, with increased economic and social costs in parallel to financial cuts in the health sector worldwide, requires the proposition of new solutions to effectively and efficiently address these society demands. International collaboration is a useful tool in this context since it is recognized by increasing the capacity to tackle complex problems from a variety of perspectives, allowing identify comprehensive research issues that consider health disparities, the particularities of different health systems, just as the cultural influence in all areas of health in addition to foster the development of research skills. If well established, its results have the power to influence health treatments worldwide by properly informing healthcare policy-makers and thus contributing to the promotion and restoration of health as well as the well-being and comfort of general population, including vulnerable and marginalized groups.

Universities along with organizations and professional associations, have a key role in the promotion and management of international collaboration, because this common agenda needs to be based on and generate scientific evidence. Thus, research is at the core of these collaborations. In addition, universities have the role of educating nurses engaged in the evidence-based care with an open mind to internationalization. Through graduate study programs, promotion of partnerships including master, doctoral and post-doctoral students results in researchers with the necessary tools to establish future partnerships. Universities still have a central role to catalyze the gathering of not only researchers but also professionals, managers and users of the health system in the joint determination of priorities for action, in the evaluation of relevance and adequacy of the proposed strategies and in the appraisal of results obtained, turning them into recommendations for clinical practice.

The potential success of such a partnership depends on careful and strategic planning^1^, which includes the establishment of the expected benefits of the project as well as its feasibility from a financial point of view. Common sources of funding are possible; however, as such competitions are relatively limited and sometimes very specific, it is essential that the involved parties search for local funding. Hereafter, evaluating differences in language and culture is essential to prevent them to become barriers to effective communication. Differences between cultures such as policies, values and even the requirements and procedures concerning the ethical aspects of research should be considered and respected. The roles and responsibilities of each team member should be well defined in order to explore and maximize the potential strengths of each one and, if necessary, these roles and responsibilities must be reviewed, in order to meet the needs of the project throughout its implementation, requiring adaptability and flexibility from the team members. Some authors^2^ interestingly refer to the theoretical perspectives of Habermas and Piaget, respectively on manifestation of social relations and construction of knowledge, for the analysis of the complex interactions among the actors implied in this type of collaboration, just as its results, which should be examined in light of their significance and applicability in clinical practice, as well as its relevance in the production of knowledge. The dissemination of results of the research is mandatory as well as the evaluation of possible extensions of the project or design of new derivative studies.

In the past decade, several articles in nursing reinforce the importance of collaboration to improve the quality of scientific production and to better meet the health demands. Many universities have established projects of broad or specific international collaboration between colleges. Another way to look for and promote international collaboration is the association with research networks that aim to promote such partnerships. Not exhaustively, among the networks there are, in the European context, *The European Academy of Nursing Science* ^*^ and the *Researching Complex Interventions for Nursing* (REFLECTION)^**^, and in Canada, the *Quebec Nursing Intervention Research Network* (RRSIQ)^***^. The objectives of these networks are consistent in proposing an interdisciplinary network in nursing to stimulate and support the development and application of knowledge that meets the health needs of society. Getting to know and integrating these networks can be a special opportunity to participate in the development of innovative research with potential impact on the health of populations.

## Footnotes

* http://www.european-academy-of-nursing-science.com/

** http://www.esf.org/coordinating-research/research-networking-programmes/biomedical-sciences-med/current-esf-research-networking-programmes/researching-complex-interventions-for-nursing-reflection.html

*** http://rrisiq.com/en/home
